# Supplementary material for: Predicting visual function by interpreting a neuronal wiring diagram
Source: Nature. 2024 Oct 2;634(8032):113–23. doi: 10.1038/s41586-024-07953-5 (PMC11446822; doi:10.1038/s41586-024-07953-5)
Supplement: Supplementary file 6 — CRF and ERF predictions for individual TmY4 and TmY9 cells. Analogous to Supplementary Data 3, but for TmY target types. Shown are the top four monosynaptic pathways, the strongest pathway passing through each of the top ten intermediary types (ranking from Extended Data Fig. 7), and the trisynaptic pathway Tm1–TmY–Dm3–TmY (see the section entitled Prediction of spatial normalization). [file 41586_2024_7953_MOESM6_ESM.zip › DataS4/TmY9qΓèÑ/720575940632268940.pdf]

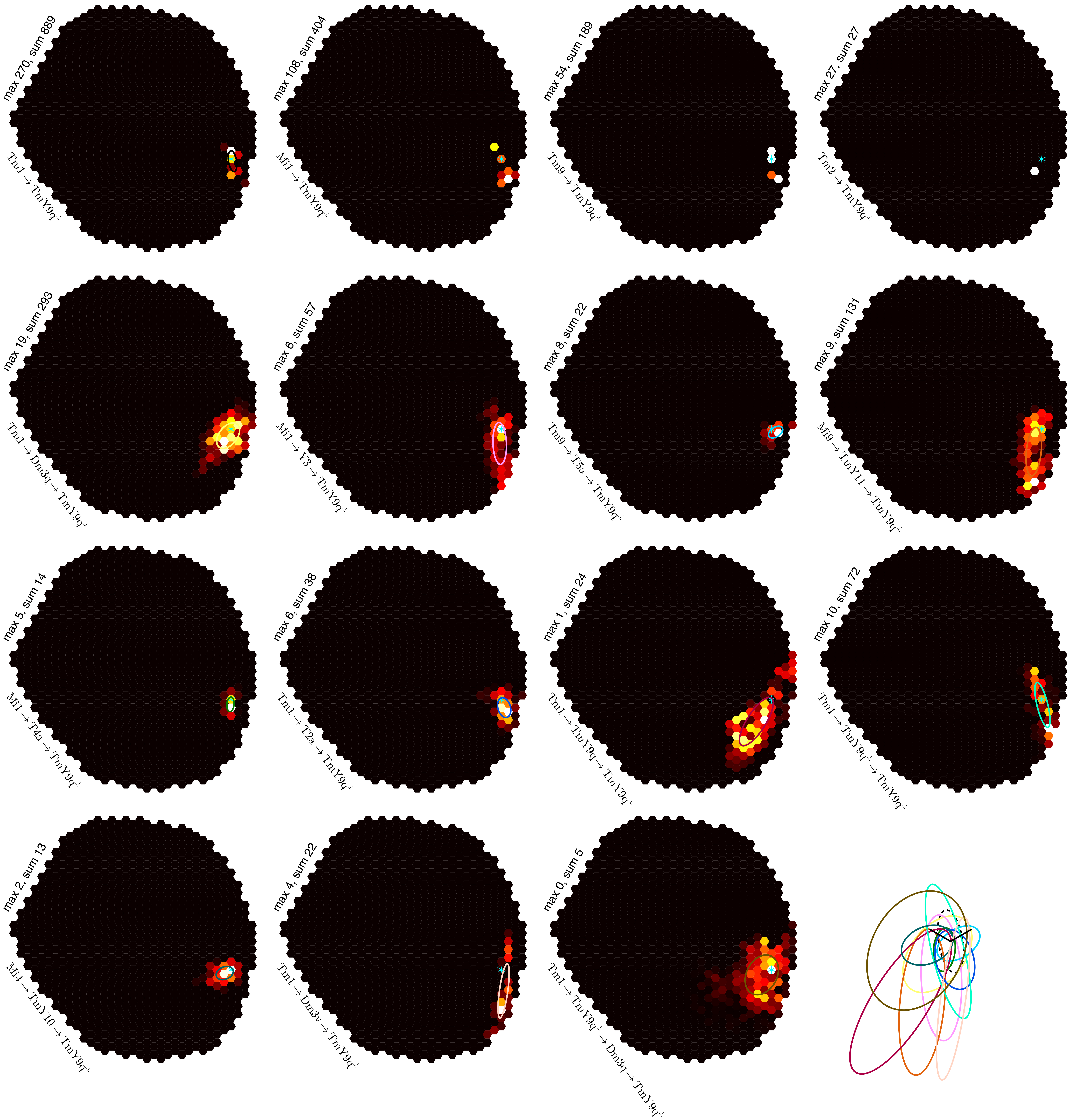
max 270, sum 889
Tm1 → TmY9q⊥
max 108, sum 404
Mi1 → TmY9q⊥
max 54, sum 189
Tm9 → TmY9q⊥
max 27, sum 27
Tm2 → TmY9q⊥
max 19, sum 293
Tm1 → Dm3q → TmY9q⊥
max 6, sum 57
Mi1 → Y3 → TmY9q⊥
max 8, sum 22
Tm9 → T5a → TmY9q⊥
max 9, sum 131
Mi9 → TmY11 → TmY9q⊥
max 5, sum 14
Mi1 → T4a → TmY9q⊥
max 6, sum 38
Tm1 → T2a → TmY9q⊥
max 1, sum 24
Tm1 → TmY9q → TmY9q⊥
max 10, sum 72
Tm1 → TmY9q⊥ → TmY9q⊥
max 2, sum 13
Mi4 → TmY10 → TmY9q⊥
max 4, sum 22
Tm1 → Dm3v → TmY9q⊥
max 0, sum 5
Tm1 → TmY9q⊥ → Dm3q → TmY9q⊥
